# Anatomically Guided Cascaded U-Net Ensemble for Coronary Artery Calcification Segmentation in Cardiac CT

**DOI:** 10.3390/bioengineering12111243

**Published:** 2025-11-13

**Authors:** Omar Alirr, Tarek Khalifa

**Affiliations:** College of Engineering and Technology, American University of the Middle East, Egaila 54200, Kuwait; tarek.khalifa@aum.edu.kw

**Keywords:** coronary artery calcification, cardiac CT, coronary CT angiography, U-Net ensemble, ResU-Net, vessel priors, Frangi vesselness, medical image segmentation

## Abstract

Accurate segmentation of coronary artery calcifications (CAC) from cardiac CT is challenged by class imbalance, small lesion size, and anatomical ambiguity. We present an anatomically guided, cascaded framework that couples heart and vessel priors with a heterogeneous U-Net ensemble for robust, vessel-aware CAC segmentation. First, a ResU-Net trained on MM-WHS isolates the heart region of interest (ROI). Second, a ResU-Net trained on ASOCA—using Frangi vesselness enhancement—segments the coronary arteries, yielding vessel masks that constrain downstream lesion detection. Third, calcifications are segmented within the vessel-constrained ROI using an ensemble of U-Net variants (baseline U-Net, Residual U-Net, Attention U-Net, UNet++). At inference, a rank-based selective fusion strategy prioritizes predictions with strong morphological consistency and vessel conformity, suppressing false positives. On the Stanford COCA gated dataset, the proposed ensemble outperforms individual models (Dice 84.25%, sensitivity 87.10%, specificity 98.00%), with ablations demonstrating additional gains when vessel priors are integrated into selective fusion (Dice 85.50%, sensitivity 88.53%). Results confirm that combining dataset-specific anatomical priors with selective ensembling improves boundary sharpness, small-lesion detectability, and anatomical plausibility, supporting reliable CAC segmentation in clinical imaging workflows.

## 1. Introduction

Coronary artery calcium (CAC) is a highly specific and sensitive marker of coronary atherosclerosis, directly reflecting the burden of calcified plaque within the coronary arteries. Decades of pathological and population studies have demonstrated a strong association between CAC detected on cardiac computed tomography (CT) and the presence, extent, and severity of coronary artery disease (CAD) [1,2,3]. Importantly, the Agatston score—quantifying the area and density of segmented CAC regions—has emerged as one of the most robust noninvasive predictors of future adverse cardiovascular events, including myocardial infarction and sudden cardiac death. Clinical trials and observational cohorts have shown that incorporating CAC scores into risk models reclassifies risk more accurately than traditional factors alone, especially for individuals with intermediate Framingham or pooled cohort risk. Consequently, major global guidelines now endorse CAC scoring for risk stratification, personalized preventive therapy decisions such as statin initiation, and longitudinal disease monitoring [4,5]. Precise, reliable segmentation of CAC from CT images is therefore indispensable for automated, trustworthy Agatston scoring and for promoting evidence-based, patient-centered cardiovascular care [6,7,8,9,10,11,12].

In this field, three public datasets have been used, COCA (Coronary Calcium and Chest CTs, Stanford AIMI), ASOCA (Automated Segmentation of Coronary Arteries challenge, CCTA with lumen masks), and MM-WHS 2017 (Multi-Modality Whole-Heart Segmentation, CT/MRI with whole-heart structures). These datasets are used consistently throughout the paper, with dataset-specific roles detailed in Section 2.1 [13,14,15].

Automated segmentation and quantification of CAC has been studied extensively in both non-contrast cardiac CT and contrast-enhanced coronary CT angiography (CCTA). Classical CAC scoring pipelines relied on intensity thresholding and region-growing methods constrained to the cardiac region. With the advent of deep learning, CNNs and U-Net–based architectures have become the dominant approach for CAC detection and segmentation. More recently, large-scale datasets such as the Stanford Coronary Calcium and Chest CTs (COCA) have enabled robust benchmarking of CAC segmentation and automated Agatston scoring across both gated and non-gated CT protocols [16,17,18].

Despite their success, single-model U-Net approaches remain vulnerable to several challenges. Technical variability across imaging protocols and scanners, differences in noise and reconstruction, and patient-specific anatomical variations often limit their generalizability. Furthermore, small, subtle, or low-density calcifications are difficult to capture, while extracardiac high-intensity structures (such as bone or valve calcifications) may be misclassified as coronary lesions. These issues lead to false positives, missed lesions, and unreliable Agatston scores, particularly in multi-center or heterogeneous datasets [19,20,21]. Addressing these limitations requires segmentation methods that combine anatomical priors with ensemble learning strategies to improve both sensitivity and specificity.

Ensemble learning offers a promising pathway by integrating predictions from multiple architectures to exploit their complementary strengths. The rank-based selective ensembling mechanism [22], ranks model outputs according to morphological consistency and estimated quality before fusion. Unlike uniform averaging, only the top-ranked predictions are weighted and aggregated, reducing anatomically implausible false positives and improving small-lesion sensitivity. Earlier works on ranking-based ensemble selection [23,24] illustrate this broader concept.

While most CAC studies have focused on single U-Net variants like residual, attention, or nested UNet++, limited work has explored their combination within an anatomically guided pipeline. At the same time, cascaded segmentation strategies, first isolating relevant anatomical regions and then zooming in on target structures, have proven effective in other domains of medical imaging. Extending these ideas to CAC segmentation motivates a novel framework that is both anatomically informed and architecturally diverse [16,25,26,27,28,29]. Specifically, this work employed several U-Net variants for calcification segmentation, including the standard U-Net [27], Residual U-Net [30], Attention U-Net [31], and U-Net++ [32].

In this work, inspired by recent work on rank-based selective ensembling proposed by Park et al., we extend this to vessel-aware segmentation of CAC. We propose a cascaded and ensemble-based framework for automated CAC segmentation that integrates region of interest (ROI) extraction, vessel-aware priors, and heterogeneous U-Net ensembles. First, a ResU-Net trained on the MM-WHS dataset isolates the heart region, reducing background noise and class imbalance. Next, coronary arteries are segmented using a ResU-Net trained on the ASOCA dataset, with Frangi vesselness filtering to enhance vessel visibility. Finally, calcifications are segmented within the vessel-constrained ROI using an ensemble of heterogeneous U-Net variants (Residual U-Net, Attention U-Net, UNet++, and Plain U-Net) trained on calcification-positive slices from the COCA dataset. A rank-based selective ensembling strategy, constrained by coronary vessel masks, fuses predictions into a refined output that reduces false positives and preserves sensitivity to small calcifications [6,20,21,33,34].

The main contributions of this work can be summarized as follows:

- Cascaded segmentation framework: We design a three-stage pipeline for CAC segmentation that progressively extracts the heart ROI, segments coronary arteries, and detects calcifications within anatomically constrained regions.
- Heterogeneous U-Net ensemble: We integrate multiple U-Net variants into a unified ensemble, leveraging their complementary representational strengths to achieve more robust lesion detection.
- Selective ensemble fusion with anatomical priors: We introduce a vessel-aware, rank-based selective ensembling strategy that reduces false positives and improves sensitivity to small and irregular calcifications.
- Demonstrated improvement over single models: Our experiments on the COCA dataset confirm that the proposed ensemble framework consistently outperforms individual U-Net variants in Dice score, sensitivity, and specificity, highlighting its clinical potential.

The remainder of this paper is organized as follows. Section 2 describes the proposed methodology in detail, including preprocessing, ROI extraction, coronary artery segmentation, and calcification ensemble segmentation. Section 3 presents experimental results, including benchmarking against individual U-Net variants, fusion strategy comparisons, and ablation studies. In addition, Section 3 discusses the findings in relation to existing state-of-the-art CAC segmentation approaches. Finally, Section 4 concludes the paper and outlines potential future directions.

## 2. Materials and Methods

**Figure 1:**
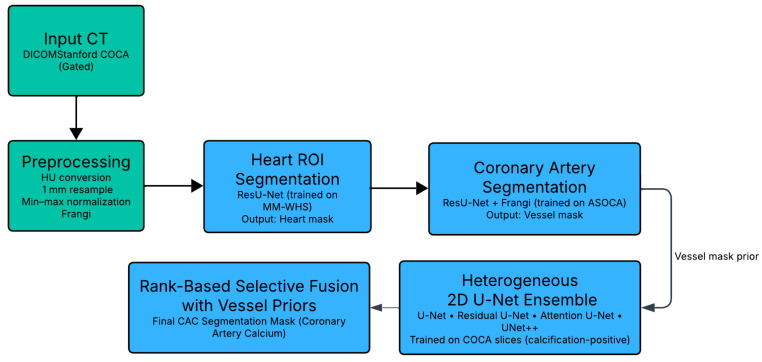
Proposed Cascaded Ensemble Framework for CAC Segmentation.

The proposed study presents a comprehensive and cascaded framework for the automated segmentation of coronary artery calcifications (CAC) from cardiac computed tomography (CT) images. The methodology integrates a multi-stage processing pipeline designed to progressively refine the analysis and enhance anatomical precision. As illustrated in Figure 1, the overall workflow consists of three major stages: preprocessing, anatomical segmentation, and calcification ensemble segmentation. The preprocessing stage standardizes CT volumes by converting pixel intensities to Hounsfield units (HU), resampling to isotropic resolution, normalizing intensities, and enhancing vessel-like structures through Frangi vesselness filtering. These operations ensure consistent image quality and accentuate coronary arteries, facilitating accurate downstream segmentation.

The cascaded segmentation stage proceeds hierarchically to capture different anatomical levels, beginning with heart region extraction, followed by coronary artery segmentation, and concluding with calcification detection. A ResU-Net trained on the MM-WHS dataset isolates the heart region of interest (ROI), while a second ResU-Net trained on the ASOCA dataset segments the coronary arteries, generating vessel priors that guide subsequent lesion detection. Finally, calcified plaques are segmented within the vessel-constrained ROI using an ensemble of heterogeneous U-Net variants; U-Net, Residual U-Net, Attention U-Net, and UNet++. Their outputs are fused using a rank-based selective ensemble strategy that prioritizes anatomically consistent predictions and suppresses false positives. This hierarchical, anatomically informed design ensures that the proposed framework achieves accurate, vessel-aware CAC segmentation, laying the foundation for automated calcium burden quantification and supporting more precise cardiovascular risk assessment.

### 2.1. Clinical Dataset Description

The proposed framework integrates three complementary public datasets—COCA, ASOCA, and MM-WHS—each serving a distinct role within the cascaded pipeline for coronary artery calcification (CAC) segmentation.

The COCA dataset is the primary resource for calcification segmentation and Agatston scoring. It includes 789 gated cardiac CT volumes with full 3D CAC masks and 214 non-gated scans with corresponding calcium scores. The gated subset provides 36,411 slices, of which 3656 slices contain annotated calcifications across the major coronary arteries (RCA, LAD, LCX, LM), yielding 6211 segmented lesions. These annotations, derived from XML files, provide the voxel-level ground truth used to train the final stage of our framework—the ensemble U-Net models for CAC segmentation. Because gated CTs are synchronized to the cardiac cycle, they minimize motion artifacts and serve as the gold standard for precise calcification delineation and Agatston quantification in our pipeline.

The ASOCA challenge dataset provides contrast-enhanced coronary CT angiography (CCTA) scans with detailed coronary artery lumen annotations, including both normal and diseased vessels. While ASOCA does not contain calcification masks, its high-quality coronary vessel segmentations are leveraged in our pipeline to train the second stage—vessel segmentation within the heart ROI. This vessel mask serves as an anatomical prior, constraining the search space for CAC to vascular regions and reducing false positives from extracardiac calcifications (e.g., in the aortic wall or valves). Thus, ASOCA complements COCA by ensuring that calcification detection is vessel-aware and anatomically consistent. The ASOCA challenge dataset comprises 20 contrast-enhanced coronary CT angiography (CCTA) scans with isotropic resolution (0.6–0.8 mm^3^) and expert-annotated coronary artery masks delineating the LM, LAD, LCX, and RCA branches. The slice size is 512 × 512 per scan.

The MM-WHS 2017 dataset provides annotated cardiac CT angiography and MRI scans with segmentation masks for key heart structures (ventricles, atria, great vessels). While it does not include calcification labels, MM-WHS is used in our framework for the first stage—heart ROI segmentation. Training on MM-WHS enables robust localization of the heart within thoracic CT, allowing us to crop a focused ROI that excludes irrelevant background (lungs, bones, soft tissues). This preprocessing step significantly reduces class imbalance and improves efficiency in downstream vessel and calcification segmentation tasks. The MM-WHS 2017 dataset contains 40 cardiac CT and 20 MRI volumes, each with manual annotations of major heart structures (LV, RV, LA, RA, AO, PA). The CT subset used here includes 20 cases with voxel spacing of 0.625 mm and typical dimensions of 512 × 512 per slice in each volume. These quantitative summaries align the dataset presentation with that of the COCA dataset.

By combining the strengths of these datasets; anatomical structure (MM-WHS), vessel priors (ASOCA), and lesion-level CAC ground truth (COCA), the cascaded framework achieves robust, anatomically consistent, and clinically meaningful segmentation of coronary artery calcifications.

### 2.2. Data Pre-Processing—Image Standardization and Enhancement

The preprocessing pipeline for coronary artery calcification (CAC) segmentation on the Stanford COCA gated dataset is designed to standardize geometry and contrast while producing voxel-accurate masks from the provided XML labels. COCA supplies gated coronary CT DICOM series together with coronary calcium segmentations and scores in XML format [13].

DICOM pixel data are first converted to Hounsfield Units (HU) using the Slope and Intercept, then intensity-windowed; CAC pipelines commonly clip to a broad CT range before normalization and enforce the Agatston convention that candidate calcification must be ≥130 HU (often also requiring a minimum projected area ≥1 mm^2^). These thresholds are standard across CAC scoring literature and are routinely adopted or mirrored in learning-based pipelines [34].

Scans are resampled to isotropic 1.0 mm voxels (linear interpolation for images; nearest-neighbor for labels) to harmonize spatial scale across subjects and scanners, a standard step in tubular-structure and coronary segmentation workflows that stabilizes model receptive fields and evaluation. To emphasize the vessel tree that hosts calcified plaques, a multiscale Hessian vesselness transform (Frangi filter) is applied. Frangi-type vesselness is widely used to enhance coronary vasculature and other tubular anatomy and has been reported in recent coronary segmentation studies and reviews [12].

**Figure 2:**
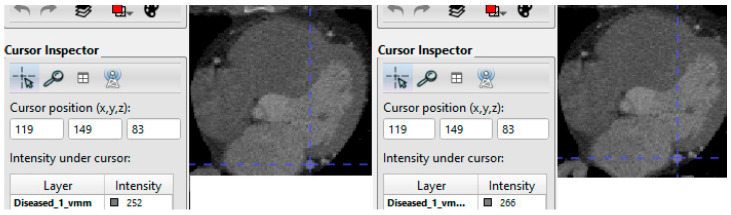
Image Preprocessing and Vesselness Enhancement; Original (**left**) vs. Enhanced (**right**).

After enhancement, intensity standardi30zation is performed—typically per-scan min–max to [0, 1] or z-score normalization—to reduce reconstruction and scanner variability before network ingestion (consistent with CAC automation studies across cardiac and chest CT), example is shown in Figure 2. These steps produce a standardized, high-contrast dataset tailored for deep learning-based CAC segmentation and quantification.

### 2.3. Segmentation of Heart and Coronary Arteries—Anatomical Prior Extraction

The second stage focuses on anatomical localization through hierarchical segmentation of the heart and coronary arteries. Accurate isolation of these regions ensures that subsequent analysis targets only relevant anatomy and reduces interference from thoracic structures such as lungs, ribs, and soft tissue. By eliminating large background areas and focusing on small vascular targets, this stage alleviates class imbalance and improves both segmentation precision and computational efficiency when processing large-scale cardiac CT data such as COCA.

**Figure 3:**
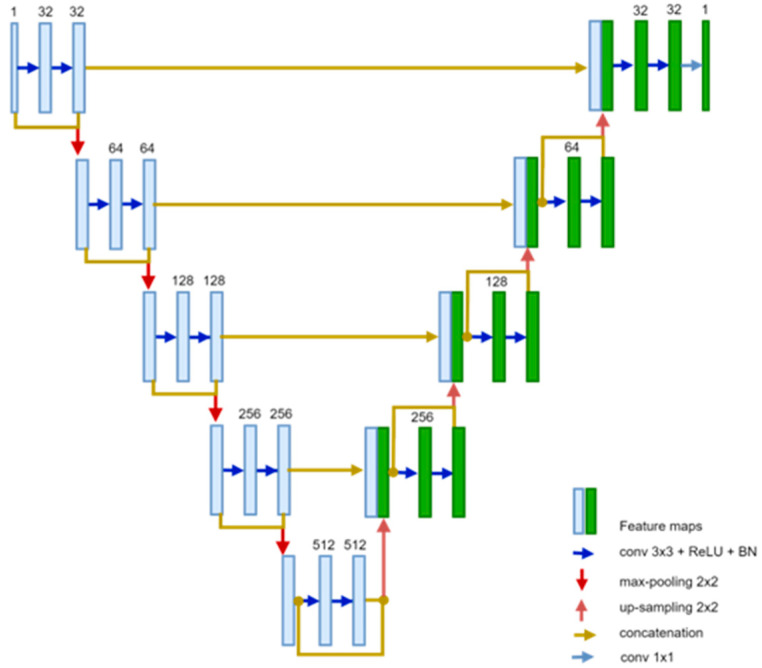
ResUnet Architecture.

To perform heart segmentation, the ResU-Net architecture shown in Figure 3 is employed due to its ability to preserve fine anatomical structures through residual skip connections while maintaining stable gradient propagation. The model is trained using the Multi-Modality Whole Heart Segmentation (MM-WHS) dataset, which provides detailed annotations of cardiac chambers and great vessels across CT and MRI modalities. Leveraging MM-WHS enables the model to learn modality-invariant representations of cardiac morphology, resulting in robust and anatomically consistent heart masks even under variations in imaging protocols or contrast levels. The trained ResU-Net is then applied to the COCA CT scans to generate precise 3D heart ROIs. These cropped ROIs form the foundation for the next two stages of the framework: coronary vessel segmentation and calcification segmentation.

Within the extracted cardiac ROI, the second stage focuses on coronary artery segmentation, which provides essential anatomical priors for vessel-aware calcification detection. For this purpose, another ResU-Net—using the same network architecture—is trained on the Automated Segmentation of Coronary Arteries (ASOCA) dataset. ASOCA offers high-resolution, contrast-enhanced coronary CT angiography (CCTA) scans with expert-annotated masks of major coronary branches, including the left main (LM), left anterior descending (LAD), left circumflex (LCX), and right coronary artery (RCA). Training on ASOCA allows the network to capture both the macro and microvascular topology of the coronary tree, supporting anatomically reliable segmentation.

To further improve vessel visibility and segmentation fidelity, a Frangi vesselness filter is applied during preprocessing. This filter computes the eigenvalues of the local Hessian matrix at multiple scales to detect tubular structures corresponding to vessels. Voxels exhibiting vessel-like curvature patterns are assigned higher vesselness scores, while nonvascular background regions are suppressed. By tuning the Gaussian scales and sensitivity parameters, both large coronary trunks and fine distal branches are enhanced, yielding an optimal vessel representation for the segmentation model.

**Figure 4:**
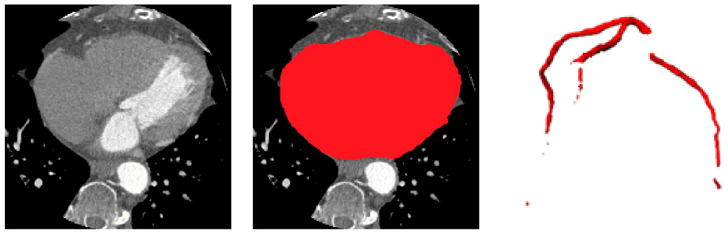
Heart and Coronary Vessel Segmentation: original CT slice, predicted heart region of interest (ROI) and 3D coronary artery segmentations.

The vesselness-enhanced inputs allow the ASOCA-trained ResU-Net to delineate coronary arteries more accurately, even in regions with low contrast-to-noise ratio. When applied to the gated COCA CT scans—cropped to the heart ROI—the resulting vessel masks serve as anatomical constraints for the downstream calcification segmentation stage. These vessel priors ensure that detected lesions are spatially restricted to plausible coronary regions, reducing false positives near the aorta, valves, or pericardium and improving the sensitivity and specificity of calcification segmentation. Examples of the heart and coronary arteries segmentations are shown in Figure 4.

### 2.4. Calcification Segmentation Using 2D Ensemble U-Nets and Selective Fusion

The final stage of the proposed framework focuses on segmenting coronary artery calcifications (CACs) within the vessel-constrained heart region of interest (ROI). Because calcifications are typically small, sparse, and irregularly shaped, full 3D training suffers from severe class imbalance—most slices contain only background, leading to biased optimization and reduced lesion sensitivity. To address this, a two-dimensional slice-based training approach is adopted, restricting learning to slices that contain calcifications in their corresponding masks. This focused design ensures that each model consistently encounters positive examples, improving sensitivity to subtle lesions and reducing the influence of irrelevant background structures.

For segmentation, an ensemble of heterogeneous U-Net variants is constructed, with each model contributing distinct representational strengths. The standard U-Net serves as a baseline, while the Residual U-Net (ResU-Net) enhances gradient stability and feature continuity through residual skip connections. The Attention U-Net introduces spatial attention gates to emphasize lesion-relevant regions, thereby improving sensitivity to small or low-contrast calcifications. UNet++ incorporates nested dense skip connections and deep supervision, facilitating multi-scale feature fusion and sharper delineation of fine boundaries. Each model is trained independently on calcification-positive slices extracted from the Stanford COCA gated dataset, which provides dense 3D annotations of coronary calcium lesions.

The rank-based selective ensembling strategy builds upon recent selective ensemble learning approaches in medical image segmentation, where model outputs are evaluated for quality before fusion rather than uniformly averaged. Unlike conventional soft-voting, which treats all models equally, this method dynamically ranks predictions according to their local reliability and anatomical plausibility. In this framework, the probability maps produced by all U-Net variants are assessed using three reliability criteria: morphological consistency (coherence and compactness of predicted calcified regions), topological continuity (smoothness and completeness of vessel-like structures), and vessel conformity (spatial alignment with the coronary vessel masks generated in Section 2.3). For each local region, ensemble members are ranked by a weighted combination of these criteria, and only the top-ranked predictions contribute to the final segmentation. Predictions that are fragmented, noisy, or anatomically implausible are automatically down-weighted or excluded.

By selectively emphasizing the most anatomically reliable and morphologically stable outputs, the proposed fusion effectively suppresses extracardiac false positives, enhances detection of small, low-contrast calcifications, and produces sharper vessel-aligned lesion boundaries. This rank-based ensemble design integrates model diversity with anatomical guidance, yielding both quantitative and qualitative improvements over uniform averaging. Incorporating vessel priors as spatial constraints further enforces anatomical realism, ensuring that detected calcifications lie within or adjacent to the segmented coronary tree while preventing misclassification of high-density aortic or valvular regions. Compared with soft-voting or mean-logit fusion, the rank-based strategy achieves higher Dice coefficients, improved sensitivity to small plaques, and maintains high specificity. The resulting slice-wise calcification masks are then reconstructed into 3D volumes aligned with the original CT scans, providing high-fidelity lesion maps suitable for downstream quantitative and clinical analyses.

All U-Net variants in this study employ a symmetric encoder–decoder structure comprising four down-sampling and four up-sampling levels (five resolution scales in total). The number of feature channels doubles with each level (64, 128, 256, 512, and 1024), followed by transpose-convolution up-sampling and corresponding skip connections for feature fusion. The Residual U-Net introduces residual blocks at each level, resulting in approximately 32.46 million parameters. The Attention U-Net, which integrates spatial attention gates to emphasize salient regions, contains 31.16 million parameters. The UNet++ architecture incorporates nested dense skip connections and deep supervision, increasing the parameter count to about 36.61 million. In comparison, the baseline U-Net includes roughly 31.03 million parameters. These details provide an objective basis for assessing model complexity and computational requirements across the different U-Net variants. The reader can find a visual representation of the U-Net, attention U-Net, and U-Net++ network architectures in Refs. [27,31,32], respectively.

### 2.5. Performance Measures

To evaluate the effectiveness of the proposed CAC segmentation framework, we employed commonly used quantitative metrics in medical image segmentation. The Dice Similarity Coefficient (DSC) was used as the primary metric to assess spatial overlap between the predicted segmentation and the ground truth mask:

(1) $$DSC=\frac{2\left|A\cap B\right|}{\left|A\right|+|B|}$$

where A and B represent the predicted and reference masks, respectively. A Dice score of 1 indicates perfect overlap.

The Sensitivity (also known as Recall) measures the ability of the model to correctly detect calcified pixels:

(2) $$Recall=\frac{TP}{TP+FN}$$

The Specificity reflects the ability of the model to avoid false positives by correctly identifying non-calcified pixels:

(3) $$Specificity=\frac{TN}{TN+FP}$$

where TP, FP, TN, and FN correspond to the number of true-positive, false-positive, true-negative, and false-negative voxels.

Together, these metrics provide complementary insights into segmentation performance: Dice captures spatial agreement, Sensitivity evaluates lesion detectability, and Specificity ensures suppression of extracardiac false positives.

## 3. Results and Discussion

This section presents both quantitative and qualitative evaluations of the proposed cascaded framework for automated coronary artery calcification (CAC) segmentation.

We first validate the anatomical segmentation components (heart and vessel extraction) that provide structural priors for the system, followed by benchmarking of individual U-Net variants and the proposed selective ensemble framework. Finally, we conduct an ablation study to analyze the impact of selective fusion and vessel priors on segmentation performance.

### 3.1. Heart and Vessel Segmentation Results

Although the primary focus of this work is the automated segmentation of coronary artery calcifications (CAC), the cascaded framework first relies on accurate localization of the heart and coronary vessels. To validate these upstream stages, we evaluated the ResU-Net models trained on the MM-WHS dataset (for heart segmentation) and the ASOCA dataset (for vessel segmentation).

As shown in Table 1, the heart ROI segmentation achieved a Dice score of 94.0% on MM-WHS, consistent with previously reported benchmarks in whole-heart segmentation. Vessel segmentation on ASOCA also demonstrated robust performance with a Dice score of 86.5%, confirming its reliability as an anatomical prior for downstream CAC detection. These results validate the robustness of the proposed cascaded design—ensuring that subsequent calcification segmentation operates within accurately localized cardiac regions, thereby minimizing error propagation and improving overall anatomical consistency.

**Figure 5:**
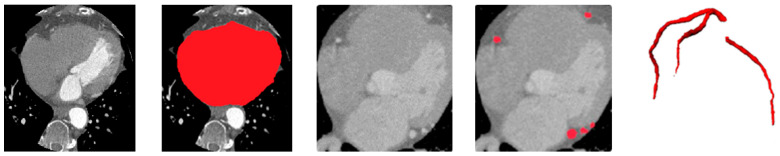
Qualitative results of heart and vessel segmentation. From left to right: original CT slice, heart segmentation, extracted heart ROI, coronary vessel segmentation, and 3D rendering of coronary vessels.

Figure 5 illustrates qualitative example of the heart and vessel segmentation results. The heart ROI masks generated by the ResU-Net trained on MM-WHS successfully isolate the cardiac region from surrounding thoracic structures. The vessel segmentation obtained from the ASOCA-trained ResU-Net clearly delineates major coronary branches, while the overlay image demonstrates precise spatial alignment of the coronary arteries within the extracted heart ROI. This confirms the anatomical plausibility and structural integrity of the upstream stages, which serve as essential priors for vessel-aware calcification segmentation in the subsequent stage.

### 3.2. Benchmark Against Base Models

Table 2 summarizes the quantitative performance of individual U-Net variants and the proposed selective ensemble on the Stanford COCA gated dataset. As expected, the plain U-Net provides the weakest baseline due to its limited capacity to capture complex contextual and multi-scale features. Among the individual variants, the Residual U-Net achieves the highest Dice score (83.3%), benefiting from residual skip connections that improve gradient flow and feature reuse, thereby stabilizing the learning process and enhancing contour continuity. The Attention U-Net and UNet++ exhibit intermediate performance: the former demonstrates stronger sensitivity to small or subtle lesions through its attention gating mechanism, while the latter leverages nested skip pathways to better integrate multi-scale context, yielding smoother and more topologically consistent boundaries.

Most importantly, the proposed rank-based selective ensemble surpasses all single-model baselines, achieving a Dice of 84.25%, sensitivity of 87.10%, and specificity of 98.00%. The ensemble consistently improves Dice by approximately +1–2% and sensitivity by +1.5% compared to the best individual model. This demonstrates that combining complementary U-Net variants within a unified ensemble effectively reduces model variance and strengthens generalization across diverse lesion morphologies and intensities. The improvement is particularly pronounced in detecting small, low-density calcifications, which are often missed by individual models. These findings align with ensemble-based medical segmentation studies, where fusing heterogeneous U-Net architectures yielded higher consistency and anatomical precision [32,35,36].

**Figure 6:**
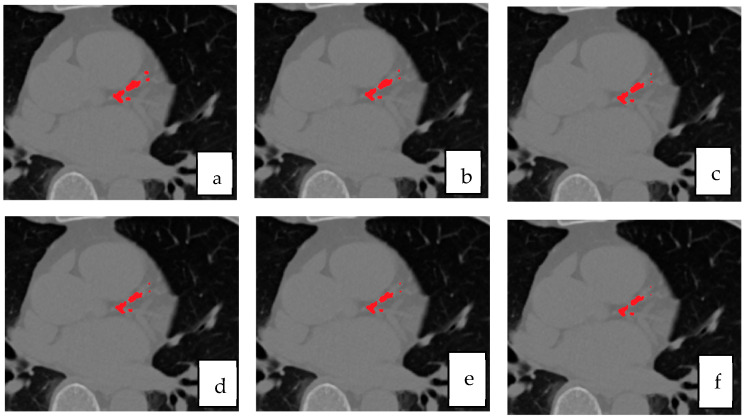
Qualitative comparison of segmentation results for coronary artery calcification (CAC). (**a**) ground truth mask, (**b**) proposed selective ensemble, (**c**) Residual U-Net, (**d**) UNet++, (**e**) Attention U-Net, and (**f**) baseline U-Net.

Figure 6 presents qualitative comparisons between the segmentation outputs of the proposed ensemble and the individual U-Net variants. From left to right, each panel shows the ground truth mask, followed by predictions from the selective ensemble, Residual U-Net, UNet++, Attention U-Net, and baseline U-Net. The visual results highlight the superior boundary delineation and reduced false positives achieved by the selective ensemble. In particular, the ensemble produces more coherent and anatomically accurate segmentation of small calcified lesions along coronary branches, while the baseline models tend to over-segment adjacent high-intensity structures such as aortic walls or valves. The qualitative evidence thus reinforces the quantitative findings, confirming that rank-based fusion not only improves numerical accuracy but also enhances clinical plausibility and visual consistency in CAC segmentation.

Although all four members of the ensemble are U-Net–based architectures, their design differences yield complementary feature representations and non-redundant error patterns. The Residual U-Net enhances continuity along vessel branches through residual skip connections; the Attention U-Net emphasizes small, low-contrast calcifications via spatial gating; and UNet++ refines multi-scale boundary delineation with dense skip pathways and deep supervision. Even the baseline U-Net contributes by producing smoother but conservative predictions that reduce variance across members. Furthermore, each model was trained with distinct random initializations, introducing additional diversity in feature learning and decision boundaries.

This combination of architectural and stochastic diversity explains why all U-Net variants contribute positively to the ensemble’s final performance, and why the improvement persists under different weighting strategies: the ensemble consistently benefits from variance reduction and complementary lesion recovery rather than from the weighting scheme itself.

### 3.3. Comparison of Fusion Strategies

To evaluate the effectiveness of the proposed rank-based selective ensembling, we compared it against the conventional soft-voting approach, where probability maps from all ensemble members are averaged voxel-wise without spatial weighting. While simple, soft-voting treats all model predictions equally, regardless of their local anatomical plausibility or consistency, often leading to over-segmentation and blurred lesion boundaries.

As shown in Table 3, the proposed selective fusion achieves higher Dice and sensitivity while maintaining high specificity. This improvement stems from the ability of the rank-based strategy to evaluate predictions based on morphological consistency, spatial continuity, and vessel conformity. By ranking each candidate prediction relative to the vessel priors obtained in the preceding stage, the method selectively emphasizes anatomically reliable regions while suppressing fragmented or false detections outside the coronary tree.

The selective ensemble improves Dice by approximately +0.55% and sensitivity by +0.6% compared to soft-voting, demonstrating that spatially guided, vessel-aware weighting is more effective than uniform averaging. This enhancement is most evident in small or low-density calcifications, where anatomical context plays a critical role in distinguishing true lesions from background noise.

Visually, the selective ensemble produces sharper and smoother lesion boundaries and exhibits fewer false positives, especially around dense extracardiac regions such as the aorta and cardiac valves.

**Figure 7:**
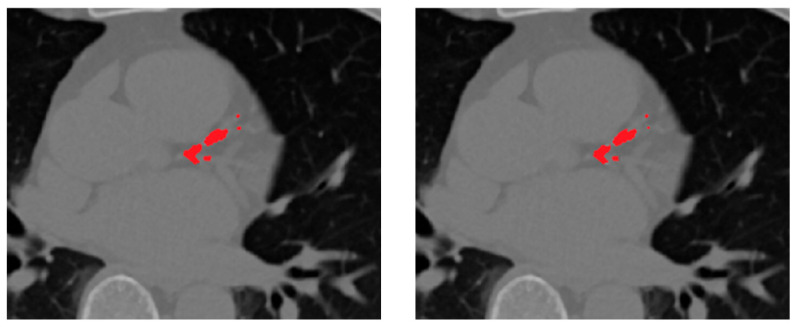
Comparison of fusion strategies for ensemble CAC segmentation. From left to right: rank-based selective ensemble output, soft-voting ensemble.

Figure 7 presents representative qualitative comparisons, illustrating that the selective fusion generates cleaner, anatomically coherent segmentation masks, effectively preserving true calcifications while suppressing spurious predictions. The proposed selective fusion produces more anatomically consistent calcification masks with reduced false positives and improved boundary continuity.

### 3.4. Ablation Study

To quantify the contribution of each component within the proposed framework, an ablation study was conducted as summarized in Table 4. Using only the best single model (Residual U-Net) serves as the baseline configuration, representing the strongest standalone learner in our architecture set. Introducing a simple soft-voting ensemble of all U-Net variants yields modest gains, reflecting the benefit of aggregating heterogeneous feature representations but still limited by uniform weighting.

Replacing soft-voting with the proposed rank-based selective fusion provides a more pronounced improvement, as the ranking mechanism prioritizes morphologically consistent, vessel-aligned predictions while suppressing isolated or anatomically implausible responses. The addition of vessel priors to this selective ensemble further refines localization by spatially constraining calcifications to the coronary tree, leading to the highest Dice and sensitivity scores overall.

As observed, the rank-based ensemble improves Dice by approximately +0.95% over soft-voting, while incorporating vessel priors adds a further +1.25% Dice and +1.4% sensitivity gain. These progressive improvements demonstrate that anatomical guidance complements ensemble diversity, yielding the most anatomically coherent and clinically reliable CAC segmentation results.

Beyond the quantitative gains, this study underscores the synergistic value of integrating anatomical priors with selective ensemble learning. The modular, cascaded design of the framework allows each stage—ROI extraction, vessel segmentation, and calcification detection—to be independently optimized or retrained for new datasets. This flexibility facilitates adaptation to other cardiac imaging modalities and multi-center data with varying contrast and acquisition parameters. In future work, we plan to extend the approach to non-gated CT protocols, enhance generalization through domain adaptation, and integrate the resulting lesion maps into comprehensive, explainable cardiovascular risk-assessment systems.

**Figure 8:**
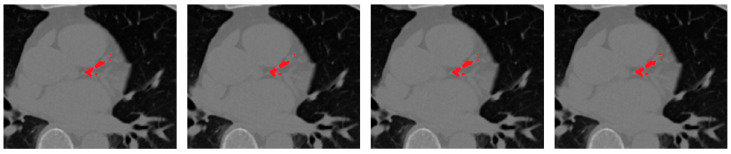
Ablation study showing the contribution of each framework component. From left to right: Residual U-Net (baseline), soft-voting ensemble, rank-based selective ensemble, and rank-based ensemble with vessel priors.

Figure 8 presents qualitative examples illustrating the progressive improvement across different configurations. The final model with vessel priors produces cleaner, vessel-aligned lesion maps and sharper calcification boundaries compared to the baseline and soft-voting ensembles. The addition of selective fusion and vessel priors progressively improves lesion sharpness, vessel conformity, and reduces false positives around extracardiac regions.

To further examine the contribution of each model within the ensemble, a leave-one-out ablation was conducted, where one U-Net variant was removed at a time and the ensemble was re-evaluated on the COCA dataset. As shown in Table 5, excluding any model decreased overall Dice and sensitivity, confirming that each variant provides complementary strengths despite sharing the same base structure. The baseline U-Net acted as a stabilizing component, mitigating over-segmentation from other members. These results demonstrate that the ensemble’s superior performance arises from architectural complementarity rather than redundancy.

### 3.5. Discussion

The results demonstrate that the proposed cascaded and ensemble-based framework significantly enhances coronary artery calcification (CAC) segmentation compared with single-model architectures. By integrating anatomical priors and a rank-based selective fusion mechanism, the framework effectively addresses two major challenges in CAC segmentation: class imbalance and anatomical ambiguity. The heart and vessel segmentation stages—trained on the MM-WHS and ASOCA datasets—provided reliable anatomical priors that restricted the search space to clinically relevant coronary regions. This anatomical constraint reduced false positives and improved lesion localization, particularly for small or low-density calcifications often missed by conventional CNN-based methods.

The ensemble stage further contributed to robustness and accuracy. Individual U-Net variants exhibited complementary strengths: the Residual U-Net enhanced gradient propagation and feature continuity, the Attention U-Net improved focus on small calcified regions, and UNet++ captured multi-scale details through dense skip connections. The proposed rank-based selective ensemble exploited these strengths by adaptively weighting predictions according to vessel-aware morphological reliability rather than uniform averaging. Consequently, the final outputs exhibited smoother lesion boundaries, reduced noise, and consistently higher Dice and sensitivity scores across the test set.

The ablation study confirmed that each architectural component contributed additively to the final performance. The inclusion of vessel priors within the selective fusion yielded the largest performance gain, emphasizing the importance of embedding explicit anatomical knowledge into data-driven models. This hybrid design—deep learning guided by domain-informed constraints—produces anatomically coherent and clinically interpretable segmentations, advancing the reliability of AI-based CAC quantification.

From a clinical standpoint, accurate and automatic delineation of coronary calcium deposits has significant value. Detailed CAC maps can enhance visualization for diagnostic evaluation and interventional planning while improving quantitative assessment of atherosclerotic burden. By constraining the analysis to the coronary tree, the framework reduces misclassification of aortic or valvular calcifications and ensures that lesion detection remains coronary-specific. Although validated primarily on gated CT datasets, the framework’s modular structure facilitates adaptation to non-gated or multi-institutional data when coupled with domain adaptation or intensity harmonization techniques.

Despite its advantages, the proposed framework still depends on supervised learning with high-quality annotations for each anatomical stage. Moreover, the 2D slice-based design, while addressing class imbalance effectively, limits global contextual understanding of calcification continuity along vessels. Future work will investigate 2.5D or lightweight 3D extensions, domain adaptation for cross-site generalization, and explainable AI (XAI) integration (e.g., Grad-CAM, Score-CAM) to enhance visual interpretability and clinical trust. These advancements aim to transition the current system into a fully automated, interpretable, and clinically deployable CAC quantification pipeline.

## 4. Conclusions

This study presented a cascaded, vessel-aware deep learning framework for automated coronary artery calcification (CAC) segmentation from cardiac CT images. The pipeline integrates anatomical localization and ensemble learning within a unified architecture to achieve robust and anatomically consistent results. A ResU-Net trained on the MM-WHS dataset first isolates the heart region of interest (ROI), followed by another ResU-Net—trained on the ASOCA dataset and enhanced using Frangi vesselness filtering—to segment the coronary arteries. These vessel masks serve as anatomical priors that constrain subsequent calcification detection to physiologically valid coronary regions.

Within this vessel-constrained ROI, calcifications are segmented using a heterogeneous ensemble of U-Net variants (Residual U-Net, Attention U-Net, UNet++, and the baseline U-Net). The proposed rank-based selective fusion, guided by vessel priors, combines the predictions of these models to suppress false positives and sharpen lesion boundaries. Experimental evaluation on the Stanford COCA gated dataset demonstrates that the selective ensemble significantly outperforms individual architectures, achieving higher Dice, sensitivity, and specificity while maintaining strong anatomical fidelity.

These findings underscore the effectiveness of combining dataset-specific anatomical priors with selective ensemble learning for reliable CAC segmentation. Owing to its modular design, the proposed framework can be readily adapted to other cardiac imaging modalities or multi-center datasets. Future research will focus on extending this framework to non-gated CT scans, improving generalization through domain adaptation, and integrating the resulting lesion maps into comprehensive, explainable cardiovascular risk-assessment systems.

## Figures and Tables

**Table 1:** Quantitative results of heart and vessel segmentation on MM-WHS and ASOCA datasets.

Task	Dataset	Dice (%)	Sensitivity (%)	Specificity (%)
Heart ROI segmentation	MM-WHS	94.0	93.0	98.0
Vessel segmentation	ASOCA	86.5	87.0	97.0

**Table 2:** Quantitative comparison of individual U-Net variants and the proposed selective ensemble on the COCA dataset.

Model	Dice (%)	Sensitivity (%)	Specificity (%)
U-Net	78.00	81.20	97.00
Residual U-Net	83.30	86.50	97.80
Attention U-Net	80.00	84.10	97.40
UNet++	81.30	85.20	97.60
Selective Ensemble	84.25	87.10	98.00

**Table 3:** Quantitative comparison of fusion strategies for ensemble CAC segmentation.

Fusion Strategy	Dice (%)	Sensitivity (%)	Specificity (%)
Soft-voting	83.70	86.5	97.8
Selective ensembling	84.25	87.10	98.00

**Table 4:** Ablation study of framework components for CAC segmentation on the COCA dataset.

Configuration	Dice (%)	Sensitivity (%)	Specificity (%)
Best single model (Residual U-Net)	83.30	86.50	97.80
Ensemble (soft-voting)	83.70	86.5	97.8
Ensemble (rank-based selective)	84.25	87.10	98.00
Ensemble (rank-based + vessel priors)	85.50	88.53	98.37

**Table 5:** Leave-one-out ablation of ensemble members on the COCA dataset.

Configuration	Dice (%)	Sensitivity (%)	Specificity (%)	Dice Difference
Ensemble (rank-based selective)	84.25	87.10	98.00	—
– Residual U-Net removed	83.30	86.20	97.85	−0.95
– Attention U-Net removed	83.00	85.80	97.90	−1.25
– UNet++ removed	83.20	86.00	97.80	−1.05
– U-Net removed	83.60	86.50	97.90	−0.65

## Data Availability

The data used in the study are publicly available in [ASOCA challenge] at [https://asoca.grand-challenge.org/ (accessed on 9 June 2025)], and [MM-WHS: Multi-Modality Whole Heart Segmentation] at [https://zmiclab.github.io/zxh/0/mmwhs/ (accessed on 9 June 2025)].
